# Book Review

**Published:** 1946

**Authors:** 


### Reviews of Books

**Cardiovascular Disease in General Practice.** By TERENCE EAST, D.M., F.R.C.P. Second Edition. Pp. x, 198. Illustrated. London: H. K. Lewis & Co. Ltd. 1946. Price 12s. 6d. net.—In this little book Dr. East has succeeded in presenting the essentials of cardiology. The style is clear and concise and it is noteworthy that, despite the addition of new material, the new edition is actually shorter than the first. Throughout emphasis is laid on the importance of accurate diagnosis, if treatment is to be satisfactory. The simple clinical methods by which this accuracy can be attained are clearly described. Written as it is for the general practitioner, electrocardiograms and special methods of investigation are excluded, but the author shows how, by the proper use of knowledge gained by such special methods, they can largely be dispensed with in ordinary practice. Possibly the section on the diagnosis of the normal heart could have been expanded with value, since this is often of such great difficulty to the practitioner. However, this is perhaps a small matter, and the book can be thoroughly recommended both for careful reading and for ready reference.

**The Nervous Child.** By HECTOR C. CAMERON, M.A., M.D., F.R.C.P. Fifth Edition. Pp. viii., 252. Illustrated. London: Oxford University Press (Geoffrey Cumberlege). 1946. Price 10s. 6d.—The appearance of a fifth edition of this book, without the introduction of any great changes since its first appearance more than twenty-seven years ago, speaks well for its popularity. No one could call it an erudite enquiry into the fundamental causes of nervousness and difficulties in behaviour of children, and it is because of its simplicity and practical directness that so many people continue to like it. It is, in fact, an excellent first-aid book, and children are on the whole so adaptable, so biddable and so suggestible that first-aid is all that most of them want. It is doubtful if plain instruction and suggestion is so effective in the treatment of the parent which the author agrees is so often necessary, but this pleasantly-written little volume does not set out to give instructions in the treatment of adults. The author believes that the chief psychological characteristics of the child are its imitativeness, suggestibility, love of power and reasoning faculties within its own limited and rather peculiar scope, and its behaviour can be understood and treated along these lines. He believes that the value of Freud's teaching is in understanding our own behaviour rather than that of the child, but he shows little signs of either understanding or sympathy with Freud's major theories. He relies on suggestion and persuasion in treatment and stresses the importance of the psychosomatic relationship with particular emphasis on acidosis and hypoglycæmia on the physical side. For the treatment of minor psychological ailments of children this book will be a great help both to doctors and parents.

**Cancer of the Scrotum in Relation to Occupation.** By S. A. HENRY, M.D., F.R.C.P., D.P.H. Pp. viii., 112. Illustrated. London : Oxford University Press (Geoffrey Cumberlege). 1946. Price 15s. net.—This is careful analysis of notified cases of the disease, and another of fatal cases, from 1920 to 1943 : to show the effect of certain specific agents in causation. It begins with an account of the disease itself, from the clinical aspect and a historical summary, and contains many illustrations. It seems a pity that only a paper cover is provided instead of a binding more adapted to withstand frequent handling for reference.

**A Handbook of Radiography.** By JOHN A. ROSS, M.A., M.R.C.S., L.R.C.P., D.M.R.E. Second Edition. Pp. viii., 160. Illustrated. London : H. & K. Lewis Co. Ltd. 1946. Price 10s. 6d.—This is essentially a book for the use of radiographers in training. It is well produced and contains much information of use to radiographers when used as a minor book of reference. There are some useful formulæ in the appendix at the end of the book. It could, however, be much improved were line drawings, showing the salient features of the radiographs taken in each of the views described, included as text-book figures, together with the line drawings already provided showing the set-up of the patient. Some of the elementary technical details included in the early chapters in the book could with advantage be omitted, as the radiography student needs to know much more about the scope of these subjects than is indicated in this book. More detailed information on the care of the casettes and apparatus would be useful. A full account of the preparation of the surgeon's table, with reference to the aseptic technique for special examinations such as intravenous examinations and myelography could with advantage be included.

**Theory and Practice of Nursing.** Fifth Edition. By M. A. GULLAN. Pp. vii., 242. Illustrated. London : H. K. Lewis & Co. Ltd. 1946. Price 12s. 6d.—Miss Gullan's book has always been a popular and helpful text book. It is, as explained in the preface, " suggestive rather than exhaustive in character." This new edition should be equally helpful to the student-nurse. It is surprising to find the word probationer still used. There are many very practical suggestions in nursing procedure, but some appear to be somewhat vague, *e.g.*, (*a*) The preparation of a Turpentine Enema. (*b*) The " Witness " ? to a Nasal Feed. (*c*) A " soft handkerchief " placed under a leech. It would seem that several sections have not been brought up to date, *e.g.*, (*a*) Croton oil to diminish arterial pressure. (*b*) Glass Catheters are advocated. (*c*) " Antiphlogistine," a " proprietary preparation " is mentioned instead of Cataplasma Kaolin. (*d*) Enteric Fever is not now nursed in a " general ward." No mention is made of the more modern methods of feeding such patients. Surely it is not good to tell nurses to chip ice with a " Cap pin " !

